# Impaired Endothelium-Mesenchymal Stem Cells Cross-talk in Systemic Sclerosis: a Link Between Vascular and Fibrotic Features

**DOI:** 10.1186/s13075-014-0442-z

**Published:** 2014-09-24

**Authors:** Paola Cipriani, Paola Di Benedetto, Piero Ruscitti, Antonio Francesco Campese, Vasiliki Liakouli, Francesco Carubbi, Ilenia Pantano, Onorina Berardicurt, Isabella Screpanti, Roberto Giacomelli

**Affiliations:** Department of Applied Clinical Sciences and Biotechnology, Rheumatology Unit, School of Medicine, University of L’Aquila, Delta 6 Building, Via dell’Ospedale, 67100 L’Aquila, Italy; Department of Molecular Medicine, School of Medicine ‘Sapienza’ University of Rome, Viale Regina Elena, 324, 00161 Rome, Italy

## Abstract

**Introduction:**

To assess if an impaired cross-talk between endothelial cells (ECs) and perivascular/multipotent mesenchymal stem cells (MSCs) might induce a perturbation of vascular repair and leading to a phenotypic switch of MSC toward myofibroblast in Systemic Sclerosis (SSc).

**Methods:**

We investigated different angiogenic and profibrotic molecules in a tridimentional matrigel assay, performing co-cultures with endothelial cells (ECs) and bone marrow derived MSCs from patients and healthy controls (HC). After 48 hours of co-culture, cells were sorted and analyzed for mRNA and protein expression.

**Results:**

ECs-SSc showed a decreased tube formation ability which is not improved by co-cultures with different MSCs. After sorting, we showed: i. an increased production of vascular endothelial growth factor A (VEGF-A) in SSc-MSCs when co-cultured with SSc-ECs; ii. an increased level of transforming growth factor beta (TGF-β) and platelet growth factor BB (PDGF-BB) in SSc-ECs when co-cultured with both HC- and SSc-MSCs; iii. an increase of TGF-β, PDGF-R, alpha smooth muscle actin (α-SMA) and collagen 1 (Col1) in both HC- and SSc-MSCs when co-cultured with SSc-ECs.

**Conclusion:**

We showed that during SSc, the ECs-MSCs crosstalk resulted in an altered expression of different molecules involved in the angiogenic processes, and mainly SSc-ECs seem to modulate the phenotypic switch of perivascular MSCs toward a myofibroblast population, thus supporting the fibrotic process.

## Introduction

Systemic slcerosis (SSc) is an autoimmune disease characterized by early vascular abnormalities and subsequent fibroblass activation to myofibroblasts, leading to fibrosis of the skin and internal organs [1,2]. Much effort has been made in recent years, to identify the earlier clinical markers of SSc, to diagnose patients in a very early phase before the occurrence of fibrosis [3]. Similarly, much effort has been made to identify the earlier pathogenetic steps that may be able to activate fibroblasts after the endothelial injury [4]. Recently, many studies have pointed out the role of endothelial cells/pericytes interactions in leading fibrosis after vascular injury, during different fibrotic disorders [5,6], and these models open new perspectives to understand the fibrotic process in SSc. At present, it is well-established that at the onset of the disease a microvascular injury, associated to both ischemia/reperfusion damage and impaired compensatory angiogenesis [7,8], results in a capillary rarefaction with consequent tissue ischemia. The complex link between vessel disappearance and fibroblast activation is still largely unknown. It is well-known that the maintenance of the existing vasculature and the stabilization of the newly formed vessel is successfully achieved by strict interplay between endothelial cells (ECs) and pericytes, mediated by both direct contact and orchestrated actions of cytokines and growth factors [9].

During the normal angiogenic process, mesenchymal pericyte progenitors are recruited in the vascular sprouts in response to growth factors, mainly PDGF, which is produced by ECs [10]. The contact between ECs and perivascular progenitors is a pivotal step for inducing ECs to produce active transforming growth factor-β (TGF-β) [11], which is able to induce maturation of mural cells [12,13], promoting blood vessel stabilization and function [14]. Finally, vascular endothelial growth factor (VEGF), produced by mesenchymal stem cells (MSCs), makes ECs unresponsive to other proangiogenic stimuli.

Several studies provide evidence that, during adult life, resident pericytes, distributed in different tissues, represent the local sources of stem cells. In fact pericytes may differentiate toward a variety of mesenchymal populations, such as osteoblasts, chondrocytes, adipocytes and fibroblasts [15], supporting the concept that adult multipotent MSCs are members of the pericyte family and reside in a specialized physical location known as the perivascular niche [16]. In this place, MSCs regulate their own fate by the heterotypic cell-cell crosstalk with ECs [17]. This contact maintains MSCs in their quiescent state or, alternatively, provides signals activating their differentiative process [18]. This phenotypic modulation is a critical event, which may lead to myofibroblast differentiation, resulting in abnormal expression of contractile proteins, such as alpha-smooth muscle actin (α-SMA), and in collagen 1 (Col1) production. The pericyte-myofibroblast transition seems to be involved in many different diseases. As shown in experimental models of kidney fibrosis, altered pericyte-EC crosstalk, after microvascular injury leads to pericyte detachment, migration, and differentiation toward myofibroblasts with consequent destabilization and loss of capillaries [19,20]. Furthermore, in different experimental models, spinal cord pericytes are identified as the main progenitor population of scar tissue in the central nervous system, intestinal pericytes are the main source of myofibroblasts, in different models of colitis, and hepatic stellate cells are the major precursors of myofibroblasts in liver disease [21-23].

Recently, different authors have shown that bone marrow-derived MSCs express pericyte markers and cooperate with ECs to form a vascular network in three-dimensional cultures, suggesting that these cells may derive from the bone marrow perivascular sites where they probably act as pericytes [24,25]. From a translational point of view, the data obtained in these models on the role of perivascular cells in the fibrotic process and their possible involvement in the pathogenesis of SSc, are intriguing and until now are largely unexplored.

We recently showed that MSCs from SSc patients express pericyte-specific markers and behave as pericytes. They display an antiangiogenic and myofibroblast-like phenotype, with increased production of contractile fiber-associated proteins, probably due to micro-environmental cues operating during the disease [26].

During SSc, the fibrotic process is strongly mediated by the effect of several cytokines, including TGF-β and platelet-derived growth factor (PDGF), and elevated levels of VEGF have been reported, despite lack of compensative angiogenesis. Of note, it is well-known that the same molecules are involved in vessel formation and stabilization. Thus, a perturbation of the normal crosstalk between ECs and perivascular/MSCs in SSc might induce an abnormal production of these molecules, inducing vessel instability and perivascular-myofibroblast phenotypic transition and activation, starting the pathogenic process leading to the classical hallmarks of the disease: the diffuse fibrosis, on one hand, and the loss of peripheral vascularization on the other. The aim of the present study was to assess these specific molecular pathways during the EC-MSC crosstalk in patients with early diffuse SSc, in whom the occurrence of fibrosis is generally earlier and rapidly progressive, when compared to the limited form. We performed a three-dimensional angiogenic assay, co-culturing ECs and MSCs from SSc patients and healthy controls (HCs). After co-culture, cells were sorted for molecular- and protein-level analyses. This is the first report in the scientific literature providing evidence that ECs in SSc may induce a pathogenic phenotype in MSCs, switching from architectural and metabolic supporting cells to cells with strong migratory and profibrotic functions, thus potentially modulating vessel instability on one hand, and different pathways involved in the fibrotic process on the other hand.

## Methods

### Isolation, culture, immunophenotyping and differentiation of MSCs

After approval from San Salvatore University Hospital ethics committee and written informed consent from patients, MSCs were obtained from 10 SSc patients with the early diffuse cutaneous form of the disease (disease duration less than 3 years calculated since the first non-Raynaud’s symptom of SSc) [27], by aspiration from the posterior superior iliac crest and cultured and characterized as previously described [28]. Demographic and clinical characteristics of the patients are shown in Table 1.

**Table 1:** **Clinical and demographic features of the 10 patients with early diffuse SSc**

**Sex/age (years)**	**Year of SSc onset/disease duration at skin-BM biopsy (years)**	**MRSS/score at skin biopsy**	**Autoantibodies**	**Lung involvment HRCT/PFT**	**Heart involvement/scleroderma renal crisis**	**Raynaud’s phenomenon/digital ulcers**
F/46	2010/2	12/2	ANA/Scl-70	Normal/Normal	Normal/No	Yes/No
F/21	2009/3	13/1	ANA/Scl-70	Normal/Normal	Normal/No	Yes/Yes
F/31	2011/1	13/2	ANA/Scl7-0	Normal/Normal	Normal/No	Yes/Yes
F/36	2010/2	11/2	ANA/Scl-70	Normal/Normal	PAH/No	Yes/Yes
M/20	2010/2	11/1	ANA/Scl-70	Normal/Normal	Normal/No	Yes/No
F/41	2010/2	15/2	ANA/Scl-70	Normal/Normal	Normal/No	No/No
F/30	2010/2	10/1	ANA/Scl-70	Normal/Normal	Normal/No	Yes/No
F/21	2010/2	09/1	ANA/Scl-70	Normal/Normal	Normal/No	Yes/No
F/31	2009/3	14/1	ANA/Scl-70	Normal/Normal	Normal/No	Yes/No
F/42	2009/3	16/2	ANA/Scl-70	Fibrosis/Normal	Normal/No	Yes/No

M, male; F, female; MRSS, modified Rodnan skin thickness score (maximum possible score 51); HRCT, high-resolution computed tomography; PFT, pulmonary function test; ANA, antinuclear antibodies; ACA, anti-centromere antibodies; scl-70, anti-topoisomerase antibodies; PAH, pulmonary arterial hypertension.

Patients discontinued corticosteroids, oral vasodilators, intravenous prostanoids or other potentially disease-modifying drugs, at least one month before biopsies. None were assumed to be taking immunosuppressants.

Ten frozen bone marrow (BM)-MSCs samples obtained from age-matched healthy women who were bone marrow donors were used as control. MSCs from both SSc patients and HCs were plated at a concentration of 2 × 10^5^ cells/cm^2^ in D-MEM (GIBCO, Carsbald, CA, USA) supplemented with 10% FBS (Standard South America origin, Lonza, MD, USA), 2 mmol/L L-glutamine (EuroClone, Milano, Italy) and 100 U Penicillin, 1,000 U Streptomycin (Biochrom AG, Miramar, Fl, USA). At 80% confluence the BM-MSCs were split and sub-cultured. Third-passage (P3) BM-MSCs were analyzed for the surface expression of MSCs antigens (CD45, CD73, CD90, CD34, CD79a, PDGFRβ) and pericyte markers (α-SMA, SM22α, NG2, desmin, RGS5) as previously described [24].

### Endothelial cell isolation and culture

Microvascular ECs were isolated from skin biopsies from the same 10 SSc patients and the same 10 bone marrow donors, who kindly offered a skin sample for research purposes, after written informed consent. Biopsy samples (1 × 0.5 cm) of the involved forearm skin (skin score 1/2 at the biopsy site) were washed with PBS (Life Technologies, Van Allen Way Carlsbad, CA, USA) and four explants were placed into a 50-mL tube containing 15 mL of trypsin (Sigma-Aldrich, MO, USA) and then to digest for 45 minutes at 37°C. Cells were cultured in EGM2-MV (Lonza, Walkersville, MD, USA) at 37°C in a humidified atmosphere of 5% CO2.

Before the cells reached confluence after approximately 1 week, the heterogeneous pool of cells was exposed to a CD31-positive selection, performed with the Dynabeads magnetic CD31 MicroBeads cell sorting system (Invitrogen, Life Technologies, CA, USA). The beads rapidly target and partially coat the endothelial cells expressing the CD31 receptor.

**Figure 1:**
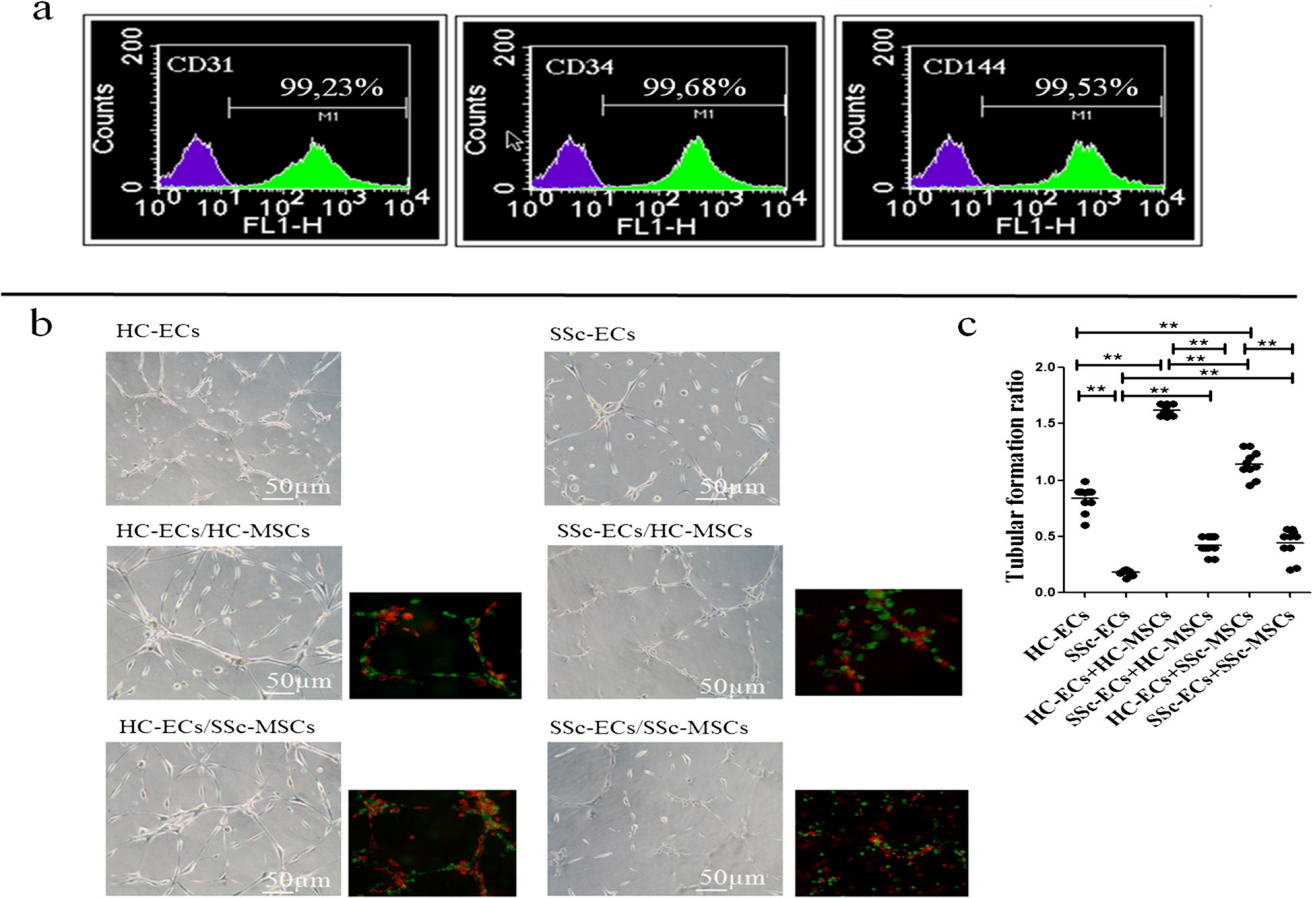
**Tubular-like structure formation in Matrigel after 48 hrs. (a)** Expression of endothelial cell markers CD31 (PECAM-1), CD34, CD144 by flow cytometric analysis. The histograms in each graph show the increase in fluorescence for each endothelial cell marker (green line) compared with the expression of an isotype-matched irrelevant monoclonal antibody (mAb) used as control (purple line). **(b)** Phase contrast pictures: healthy control (HC)-epithelial cells (ECs) cultured alone form well-organized tube-like structures; systemic sclerosis (SSc)-ECs cultured alone failed to form well-organized tube-like structures; HC-ECs/HC-mesenchymal stem cells (MSCs) co-culture: the picture shows an increased ability to form a well-organized tube-like network; SSc-ECs/HC-MSCs co-culture: a decreased capacity of HC-MSCs to improve the tubular formation by SSc-ECs when cultured alone can be observed; HC-ECs/SSc-MSCs co-culture: the presence of SSc-MSCs did not influence the ability of HC-ECs to form normal tubular structures, when cultured alone; SSc-ECs/SSc-MSCs co-culture: the impairment to form tubular structures of SSc-ECs was not improved by SSc-MSCs. The fluorescent images show EC (green) and MSC (red) distribution. Pictures are representative of all experiments. Original magnification 20×. **(c)** The tube formation was measured as cells/mm and the performance in each experiment was expressed by the ratio: total tube length in each culture condition/length in the culture of HC-ECs alone (**P = 0.0002). Results are expressed as median (range) of triplicate experiments.

After the incubation, the cells were placed in a magnet (Dynal MPC-S) (Invitrogen, Life Technologies, CA, USA) for 2 minutes, following the manufacturer’s recommended protocol for washing and final extraction. The CD31-negative cells (with no beads attached) were removed during the successive washings. The positive selected cells were 99% ECs with a specific phenotype (CD-31, CD-34, CD-144) (Figure 1a). The cells were used at the third passages.

### *In vitro* angiogenesis assay

Tube formation ability was evaluated using a Matrigel assay (Matrigel (BD Biosciences, Qume Drive San Jose, CA, USA) (8.6 mg/mL) was used at 1:1 dilution with EGM2-MV, without supplement. ECs and MSCs were labeled, before co-culture in Matrigel, using the green fluorescent dye PKH67 and red fluorescent dye PKH26 (Sigma, St. Louis, MO, USA), respectively, according to the manufacturer’s instructions. ECs and MSCs were seeded alone and in co-culture ECs/MSCs in a 3:1 ratio. The following co-cultures were performed: SSc-ECs/SSc-MSCs, SSc-ECs/HC-MSCs, HC-ECs/HC-MSCs, HC-ECs/SSc-MSCs. After 48 hrs the total tube length of each well was measured as cells/mm and photographed. Images were acquired using an Olympus BX53 fluorescence microscope.

### Cell sorting of co-cultured cells

After 48 hrs of co-culture, as suggested by available literature [29,30], tube formation was observed. At this point, MSCs and ECs were gently removed from three-dimensional cultures, using Dispase (BD Biosciences Discovery Labware, Bedford, MA, USA), and mixed with 5 mM ethylenediaminetetraacetic acid (EDTA). Dispase effectively degrades Matrigel without damaging the embedded cells. Both recovered MSCs and ECs were washed several times with PBS and re-suspended in 0.1% FBS in PBS for cell-sorting. The cells were sorted (purity >95%) by a FACSAria cell sorter (BD Biosciences, Franklin Lakes, NJ, USA) and further used for gene expression and protein profiling.

### qRT-PCR analysis

Total RNA was extracted from sorted BM-MSCs and ECs using NucleoSpin RNAXS (Macherey Nagel, Dueren, Germany) according to manufacturer's instructions and reverse-transcribed into complementary DNA (cDNA) with the ThermoScript reverse-transcription PCR system (Invitrogen, Life Technologies, CA, USA). The qRT-PCR was performed using SYBR green kits (Applied Biosystems, Life Technologies, Van Allen Way Carlsbad, CA, USA). Results were analyzed after 45 cycles of amplification using the ABI 7500 Fast Real Time PCR System. Primers were designed on the basis of the reported sequences (Primer bank NCBI; β-*actin*: 5′-CCTGGCACCCAGCACAAT-3′ (forward) and 5′-AGTACTCCGTGTGGATCGGC-3′ (reverse); α-*SMA*:5′-CGGTGCTGTCTCTCTATGCC-3′ (forward) and 5′-CGCTCAGTCAGGATCTTCA-3′ (reverse); *VEGF-A*: 5′-AGGGCAGAATCATCACGAAGT-3′ (forward) and 5′-GCTGCGCTGATAGACATCCA-3′ (reverse); *VEGFR2*:5′-GTGATCGGAAATGACACTGGAG-3′ (forward) and 5′- CATGTTGGTCACTAACAGAAGCA-3′ (reverse); *Col1A1*: 5′-AGGGCCAAGACGAAGACAGT-3′ (forward) and 5′-AGATCACGTCATCGCACAACA-3′ (reverse); *Col1A2*: 5′-TCTGGATGGATTGAAGGGACA-3′ (forward) and 5′- CCAACACGTCCTCTCTCACC-3′ (reverse); ***TGF***-β: 5′-CTAATGGTGGAAACCCACAACG-3′ (forward) and 5′- TATCGCCAGGAATTGTTGCTG-3′ (reverse); *PDGF-RR*: 5′-TGAGCGGAAACGGCTCTAC-3′ (forward) and 5′- AGTTCCTCGGCATCATTAGGG-3′ (reverse); *PDGF-BB*: 5′-CTCGATCCGCTCCTTTGATGA-3′ (forward) and 5′- CGTTGGTGCGGTCTATGAG-3′ (reverse). All gene expression data were normalized to those for β-*actin*.

### Western blot

In order to perform western blot assays, sorted BM-MSCs and ECs cells were pelleted, washed twice with PBS, lysed on ice in lysis buffer (1% Triton X-100, 0.5% NP-40, 50mMTris–Cl, pH 7.5, 150 mMNaCl, 1 mM EDTA, supplemented with 1 mMphenylmethylsulfonyl fluoride, 1 mMNaF, 1 mM Na_3_VO_4,_ 5 μg/mL aprotinin, 5 μg/mL leupeptin) for 30 minutes and cleared by centrifugation. The protein concentration was calculated by Bradford protein assay reagent (Bio-Rad Laboratories S.r.l., Segrate, MI, Italy): 50 μg of proteins were separated by SDS-PAGE and transferred to nitrocellulose membranes. After 1 hr at room temperature in blocking buffer (5% non- fat milk in Tris-buffered saline/1% tween 20 (TBS/T)) the membranes were washed three times for 5 minutes each in TBS/T, and incubated overnight at 4°C with the primary antibodies: VEGFR-2 (Invitrogen Corporation, CA, USA), VEGF-A, PDGF-BB and TGF-β (Abcam, MA, USA), PDGF-R, Col1A1 and α-SMA (Santa Cruz, Biotechnology, Dallas, Texas, USA), diluted in 5% bovine serum albumin in TBS/T. Following three washes with TBS/T, horseradish peroxidase-conjugated secondary antibodies (Santa Cruz, Biotechnology) diluted in blocking buffer was added for 30 minutes at room temperature and washed three times with TBS/T. The detection was performed by enhanced chemiluminescence detection ECL reaction (Amersham Pharmacia Biotechnology Piscataway, NJ, USA). All the signals were quantified by normalizing to the tubulin signal (CP06 Anti-α-Tubulin Mouse mAb -DM1A). The levels of proteins, in HC-MSCs cultured alone, were set to 100%; and all the results were normalized to this value. Immunoreactive bands were quantified with densitometry using ImageJ software (NIH, Bethesda, MD, USA).

### Cytofluorimetric analysis

After sorting, HC- and SSc-MSCs were incubated with the following conjugated monoclonal antibodies: allophycocyanin (APC)-conjugated monoclonal antibodies, including anti-human CD140b (PDGFRb) (BioLegend, San Diego, CA, USA) and anti-human CD309 (VEGFR2) (Miltenyi Biotec, San Diego, CA, USA ). Each fluorescence analysis included appropriate APC-conjugated negative isotype controls.

### Enzyme-linked immunosorbent assay

The concentration of VEGF-A, TGF-β and PDGF-BB released in single cultures and in co-culture supernatants were determined by ELISA using Quantikine Human Immunoassay kits (all by R&D Systems, Minneapolis, MN, USA), according to the manufacturer’s protocol. After 48 hrs of co-culture and tube formation, media samples were collected and tested.

### Statistical analysis

GraphPad Prism 5.0 software was used for statistical analyses. Results are expressed as median (range). Due to the non-parametric distribution of our data the Mann-Whitney *U*-test was used as appropriate for analyses. Statistical significance was expressed by a *P*-value ≤0.05.

## Results

### SSc-ECs affect the MSCs skill to support tubulogenesis

In our *in vitro* matrigel assay HC-ECs cultured alone formed organized tube-like structures. When these cells were co-cultured with HC- and SSc-MSCs, we observed a significant improvement in tube-formation ability without a statistically significant difference. On the contrary, as shown in Figure 1b-c, using SSc-ECs a significant impairment in tube formation was observed.

### Impaired production of angiogenic and fibrotic molecules in SSc-ECs and SSc-MSCs sorted after 48 hrs of co-culture

#### VEGF-A/VEGFR2 expression in MSCs

**Figure 2:**
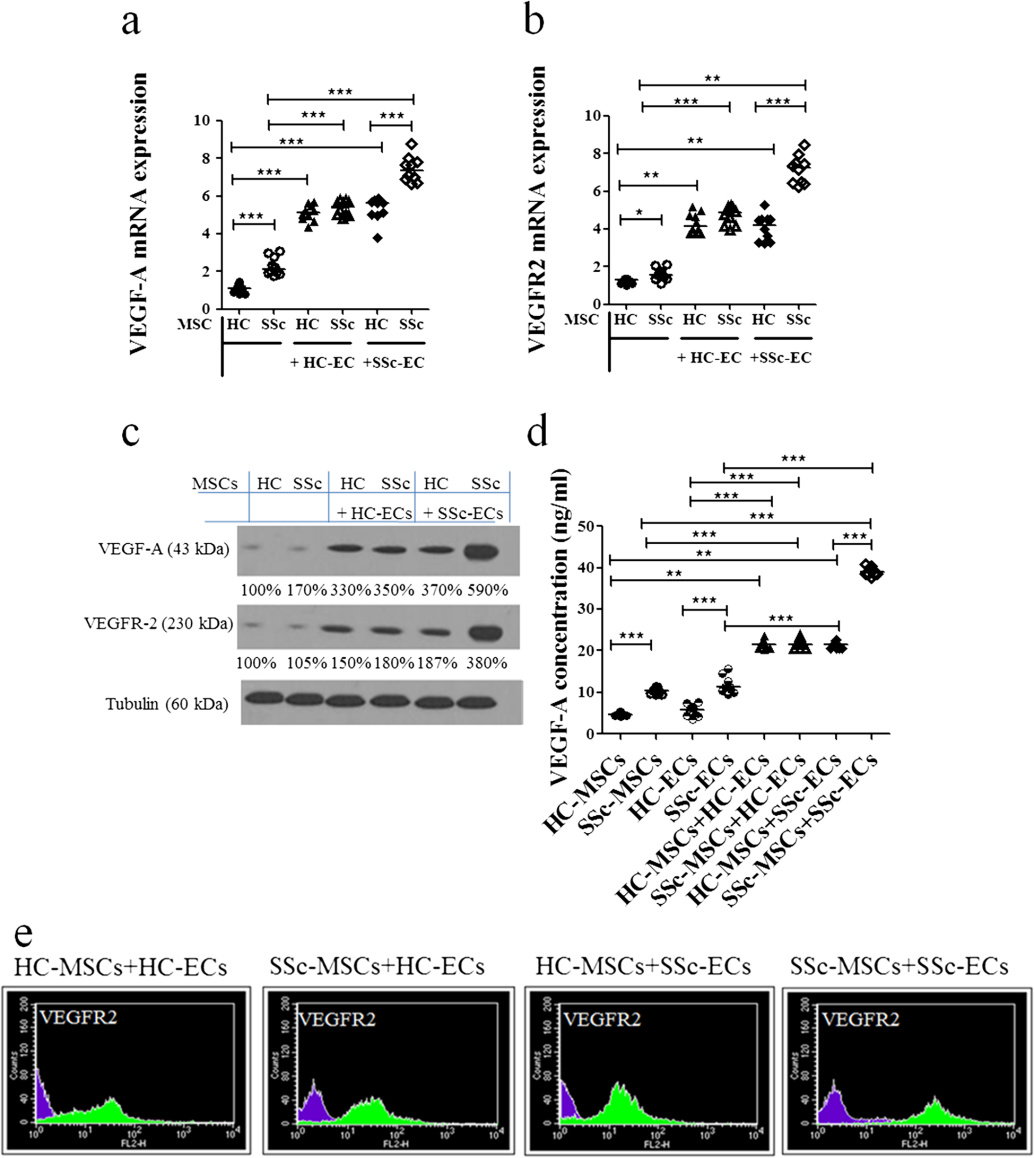
**Mesenchymal stem cell (MSC) production of angiogenic molecules when co-cultured with epithelial cells (ECs) from healthy controls (HCs) and patients with systemic sclerosis (SSc). (a, b)** The pictures show the quantification by qRT-PCR of vascular endothelial growth factor (VEGF)-A **(a)** and VEGFR-2 **(b)** mRNA levels in HC- and SSc-MSCs, sorted after co-cultures. Both SSc- and HC-ECs significantly increased VEGF-A and VEGFR-2 levels in MSCs, compared to the production from MSCs cultured alone (*P = 0.008; **P = 0.0002; ***P <0.0001). **(c)** Western blot analyses confirm results observed in qRT-PCR analyses. Pictures are representative of all experiments. **(d)** VEGF-A ELISA assays. VEGF-A was quantified in the supernatants of both single culture and co-culture conditions. The results mirrored those observed by qRT-PCR and western blot analysis (**P = 0.0002; ***P <0.0001). All the results are expressed as median (range) of triplicate experiments. **(e)** The cytofluorimetric analysis shows the fluorescence intensity of HC- and SSc-MSCs before co-culture (purple histograms) and fluorescence intensity of HC- and SSc-MSCs after co-culture (green histograms).

When MSCs were cultured alone, a statistically significant increase in *VEGF-A* transcript levels was observed in SSc-MSCs compared to HC-MSCs (2.11 (1.76 to 3.10) versus 1.11 (0.80 to 1.46), respectively, *P* <0.0001). As shown in Figure 2a the presence of SSc-ECs strongly increase the levels of *VEGF-A* mRNA transcript in SSc-MSCs when compared to the other possible mixture of cells (*P* <0.0001 for all conditions). Basal transcript levels of *VEGFR2* were significantly higher in SSc-MSCs than in HC-MSCs (1.54 (1.10 to 2.09) versus 1.28 (1.00 to 1.36), respectively, *P* <0.0088). The *VEGFR2* mRNA levels were significantly upregulated after co-culture with ECs when compared to basal conditions (*P* ≤0.0002 for both). Mirroring what was observed for the *VEGF-A*, the expression of *VEGFR2* was strongly increased in SSc-MSCs when co-cultured with SSc-ECs, as shown in Figure 2b. These results were confirmed at the protein level by western blotting analyses (Figure 2c). As far as the surface expression of VEGFR2 is concerned, as shown in Figure 2e, we observed a strong increase in intensity of VEGFR2 in SSc-MSCs when co-cultured with SSc-ECs.

Furthermore, the VEGF-A protein secreted in each culture, mirrored the results observed by qPCR and western blot analysis (Figure 2d).

#### SSc-ECs increase their expression of TGF-β and PDGF-BB, after co-culture with MSCs

**Figure 3:**
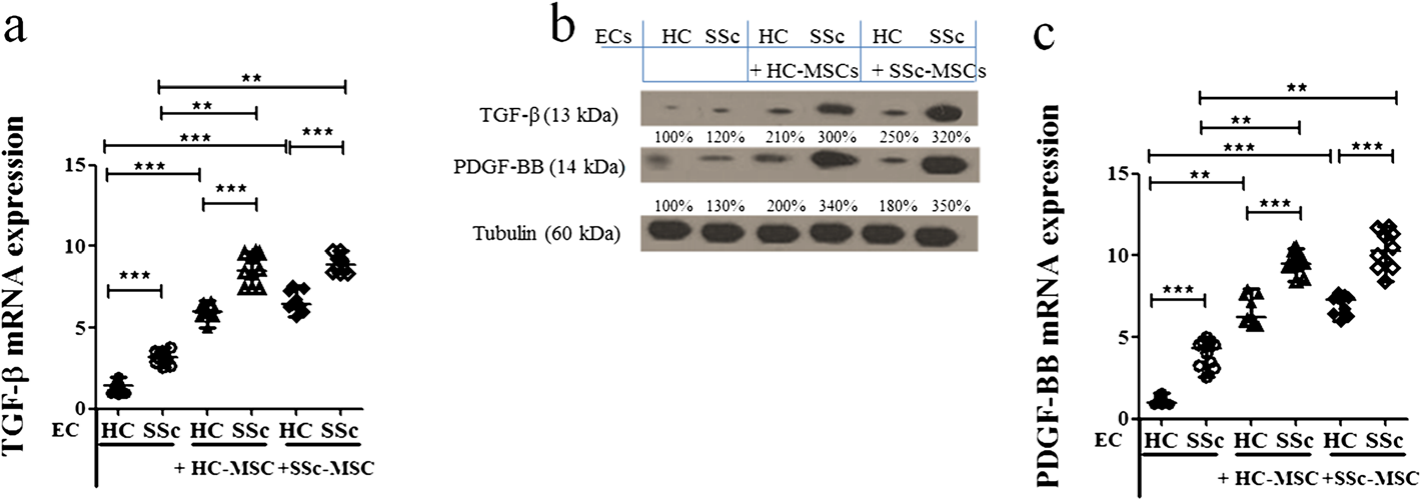
**Epithelial cell (EC) production of angiogenic molecules when co-cultured with mesenchymal stem cells (MSCs) from healthy controls (HCs) and systemic sclerosis (SSc) patients.** **(a-c)** Expression of transforming growth factor-β (TGF-β) and platelet-derived growth factor (PDGF)-BB in ECs, before and after co-culture with MSCs. SSc-ECs showed a higher amount of mRNA expression levels of both TGF-β **(a)** and PDGF-BB **(c)** compared to the expression levels of HC-ECs, when cultured alone. After co-culture with HC- and SSc-MSCs a significant increase of these expressions was observed, compared to the levels when ECs were cultured alone, the higher levels observed when SSc-ECs were in the co-cultures. All the results are expressed as median (range) of triplicate experiments (**P = 0.0002; ***P <0.0001). **(b)** Western blot analysis of TGF-β and PDGF-R protein in ECs, confirmed those observed at molecular level Pictures are representative of all experiments.

We investigated the expression of *TGF*-β in SSc-ECs and in SSc-MSCs cultured alone and after co-cultures. At the basal condition, the levels of *TGF*-β in SSc-ECs were markedly increased when compared with HC-ECs (3.18 (2.58 to 3.76) versus 1.40 (0.91 to 1.98), respectively, *P* <0.0001). When SSc-ECs co-cultured, independent of the kind of MSCs, the highest levels of *TGF*-β mRNA expression were observed, as shown in Figure 3a. These results were confirmed at the protein level by western blotting analyses (Figure 3b).

*PDGF-BB* was markedly increased in SSc-ECs cultured alone when compared to HC-ECs (4.28 (2.58 to 4.98) versus 0.98 (0.87 to 1.57), respectively, *P* <0.0001]. Mirroring the results obtained for *TGF*-β *PDGF-BB* levels were strongly increased in SSC-EC after any kind of co-culture, as shown in Figure 3c; western blotting analysis confirmed these data (Figure 3b).

#### SSc-MSCs increase their expression of TGF-β and PDGF-R after co-culture with ECs

**Figure 4:**
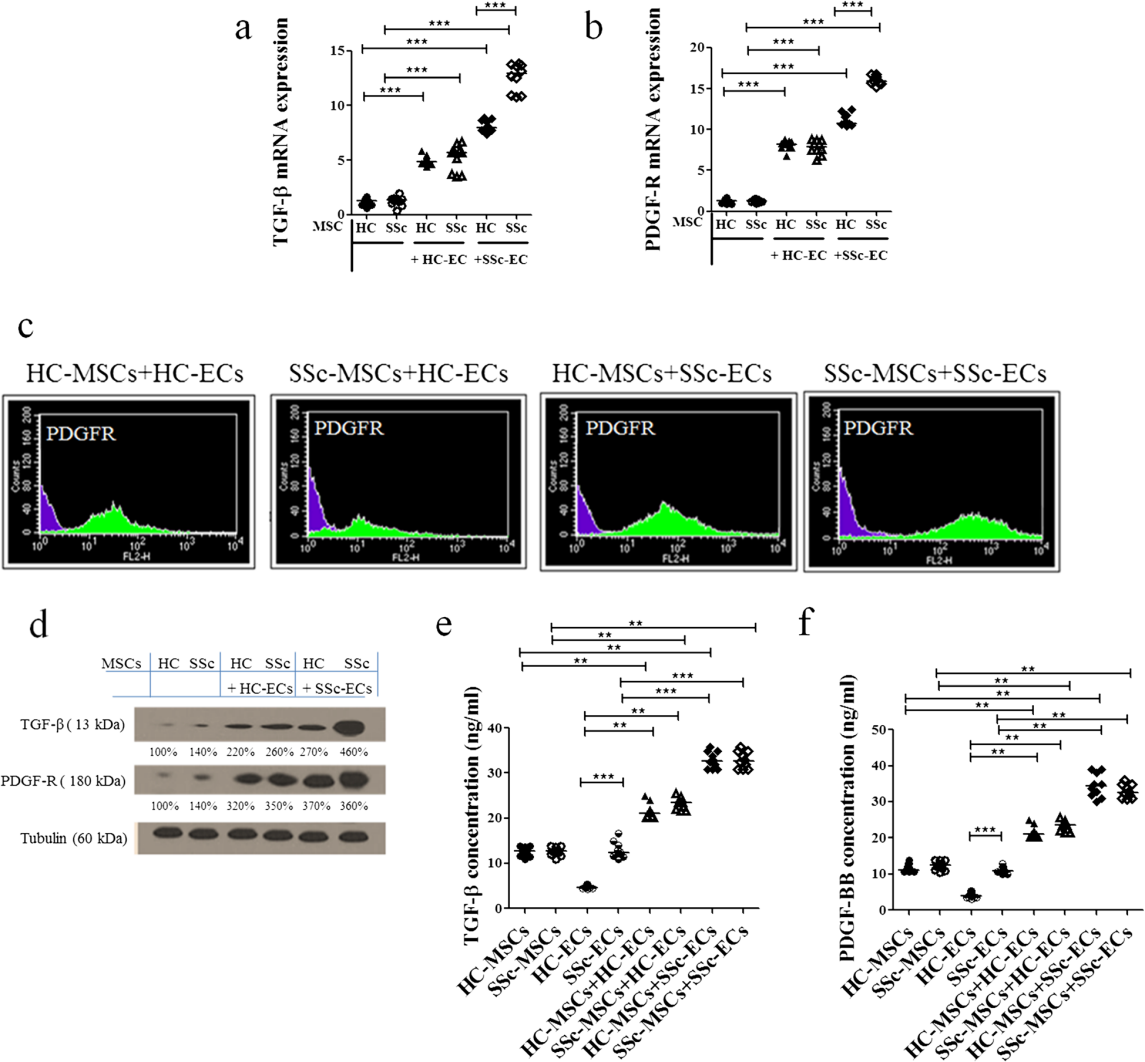
**Mesenchymal stem cells (MSCs) expression of transforming growth factor (TGF)-β and platelet-derived growth factor (PDGF)-R before and after co-culture with epithelial cells (ECs). (a, b)** MSCs from patients with systemic sclerosis (SSc) showed greater mRNA expression levels of both TGF-β **(a)** and PDGF-R **(b)** compared to the expression levels of MSCs from healthy controls (HCs), when cultured alone. Both SSc- and HC-ECs significantly increased TGF-β and PDGF-R levels in MSCs, compared to the production observed when MSCs were cultured alone, the higher levels observed in SSc-MSC/SSc-EC co-culture (***P <0.0001). Results are expressed as median (range) of triplicate experiments. **(c)** The cytofluorimetric analysis shows the fluorescence intensity of HC- and SSc-MSCs before co-culture (purple histograms) and after co-culture (green histograms). **(d)** Western blot analysis of TGF-β and PDGF-R protein in MSCs mirrored the results obtained at molecular level. Pictures are representative of all experiments. **(e, f)** TGF-β **(e)** and PDGF-BB **(f)** ELISA assays. The protein production, in the supernatant mirrored the results observed by qRT-PCR and western blot analyses (**P = 0.0002; ***P <0.0001). Results are expressed as median (range) of triplicate experiments.

*TGF*-β expression at the basal condition was similar in HC- and SSc-MSCs. As shown in Figure 4a, when SSc-ECs were added to the culture, we observed the strongest increase of *TGF*-β mRNA transcript levels in SSc-MSCs, when compared to the basal condition and when compared to the other possible mixture of cells (*P* <0.0001).

No significant difference was observed in *PDGF-R* mRNA transcripts levels between HC- and SSc-MSCs at the basal condition. We observed a significant upregulation after co-culture with ECs when compared to basal conditions. The expression of *PDGF-R* was strongly increased in SSc-MSCs when co-cultured with SSc-ECs (Figure 4b).These results were confirmed at the protein level by western blotting analyses (Figure 4d). Similarl to what was observed for *VEGFR2*, the *PDGF-R* intensity of expression was strongly increased in SSc-MSCs when co-cultured with SSc-ECs (Figure 4c).

The TGF-β and PDGF-BB quantification by ELISA showed increased release of the growth factors in the EC/MSC co-culture when compared with ECs and MSCs cultured alone (Figure 4e, f).

#### Impaired cross-talk between SSc-ECs and MSCs contribute to fibrotic switching in SSc-MSCs

Aiming to test whether the co-cultures of SSc-MSCs with ECs may contribute to SSc-MSC differentiation toward the myofibroblastic phenotype, we examined the levels of expression of α-*SMA*, *Col1A1*, and *Col1A2* in both SSc- and HC-MSCs.

**Figure 5:**
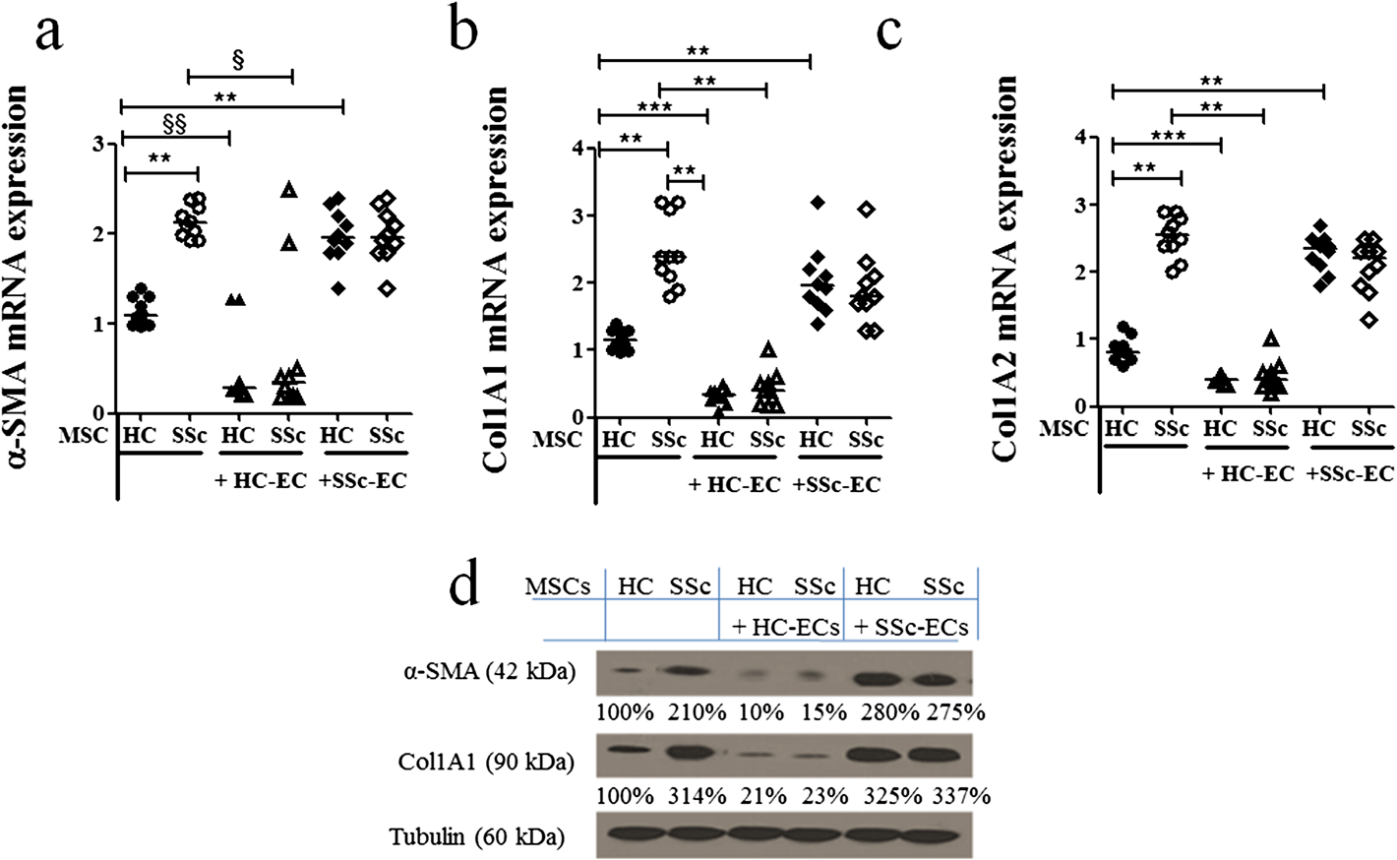
**Mesenchymal stem cell (MSC) expression of myofibroblastic molecules before and after co-culture with epithelial cells (ECs). (a, c)** When cultured alone, SSc-MSCs showed higher levels of alpha-smooth muscle actin (α-SMA) **(a)**, collagen (Col)1A1 **(b)** and Col1A2 **(c)** compared to MSCs from healthy controls (HCs) cultured alone. Co-cultures with HC-ECs, significantly decreased the mRNA levels of these genes in HC- and MSCs from patients with systemic sclerosis (SSc). On the contrary, SSc-ECs did not downregulate these gene expressions, when cultured with both HC- and SSc-MSCs (^§^ *P* = 0.0028; ^§§^ *P* = 0.0073; **P = 0.0002; ***P <0.0001). Results are expressed as median (range) of triplicate experiments. **(d)** Western blot analyses confirmed, at protein level, the results observed by qRT-PCR analyses. Pictures are representative of all experiments.

At the basal condition, SSc-MSCs showed a significant increase in mRNA levels of α-*SMA*, *Col1A1* and *Col1A2* when compared with HC-MSCs (α-*SMA* transcript levels: SSc-MSCs 2.12 (1.92 to 2.40) versus HC-MSCs 1.09 (0.97 to 1.40), *P* = 0.0002; *Co1A1* transcript levels: SSc-MSCs 2.40 (1.80 to 3.20) versus HC-MSCs 1.15 (0.97 to 1.40), *P* = 0.0002; *Col1A2* transcript levels: SSc-MSCs 2.55 (2.00 to 2.90) versus HC-MSCs 0.80 (0.60 to 1.20), *P* = 0.0002). After co-culture with HC-ECs, the SSc-MSCs displayed significant downregulation of α-*SMA* and collagen gene expression when compared to basal levels (α-*SMA*, *P* = 0.0028; *Col1A1*, *P* = 0.0002; *Col1A2*, *P* = 0.0002), as shown in Figure 5a-c. After co-culture with SSc-ECs: 1) HC-MSCs showed a significant increase of α-*SMA* and collagen-related genes when compared to their basal levels (α-*SMA*, *P* = 0.0002; *Col1A1*, *P* = 0.0002; *Col1A2*, *P* = 0.0002); 2) SSc-MSCs did not show any difference in the expression of these genes when compared to their basal conditions. Finally, the western blot analyses of α-SMA and Col1A1 mirrored the molecular results (Figure 5d).

## Discussion

This descriptive study is the first report in the available literature, providing evidence that the pathologic endothelium, via altered MSC-EC crosstalk, impairs the angiogenic process, and modulate the production of profibrotic molecules in both normal and pathologic MSCs, thus eliciting a phenotypic switch of these multipotent perivascular cells toward a myofibroblastic/profibrotic phenotype. Our data may support the hypothesis that in SSc, as shown in other pathological model of fibrosis [31], previously damaged ECs [32] may be involved in the earlier steps leading to fibrosis.

Using a tri-dimensional Matrigel system for EC/MSC co-cultures, we showed a highly significant reduction of the ability to form tubular structures when SSc-ECs were cultured with SSc-MSCs, whereas the presence of MSCs (from both patients and HCs) in each condition improved the angiogenic ability of ECs cultured alone. To better understand this finding, after 48 hrs of co-culture, we generated purified populations of cells by cell sorting. The analyses of cellular behavior after this interplay resulted in dramatic changes in gene expression and levels of proteins involved in the main steps of the angiogenic process, including the upregulation of profibrotic and extracellular matrix molecules.

In fact, as far as the production of VEGF-A, by MSCs, was concerned, the highest levels of gene expression and protein production were observed in SSc-MSCs when co-cultured with SSc-ECs, although paradoxically the same co-culture showed the worst tube-formation performance. To understand this apparent paradox, it must be pointed out that as already shown [33], perivascular cell-derived VEGF-A exerts a unique role in driving blood vessel maturation and stability, functionally distinct from tissue-derived VEGF-A, the latter promoting the vessel instability that precedes EC detachment and migration at the beginning of the angiogenic process. This physiologic switch, from tissue-derived to mural cell-derived VEGF-A, occurring at the end of the vessel maturation, fosters vessel stabilization and ultimately leads to tissue-derived-VEGF independence [33,34]. Furthermore, Greemberg *et al*. [35], demonstrated that VEGF abolished pericyte coverage of vascular sprouts, leading to vessel destabilization. VEGF-mediated activation of VEGFR2 suppressed PDGFR signaling, through the induction of a VEGFR/PDGFR complex. These data might explain our results of lower angiogenic performance, despite the increased VEGF-A production. Alternatively, a switch from proangiogenic to antiangiogenic VEGF isoforms might be considered, as recently described in SSc [36,37], although available literature shows conflicting results about the real presence and the potential inhibitory role of these antiangiogenic isoforms in human tissue [38].

Recent studies proposed an intriguing novel role for perivascular cells as a potential source of myofibroblasts in wound healing and in fibrotic disorders. Strong evidence has arisen from studies of the kidney, skin, lung, liver, and brain [39,40]. SSc, mirroring other fibrotic diseases, is characterized by the abnormal persistence of myofibroblasts, overexpressing the highly contractile protein α-SMA and producing collagen and extracellular matrix components [31]. On the bases of our results, we may hypothesize that this myofibroblast differentiation reflects a phenotypic switch of multipotent perivascular cells in the disease. It has been suggested that these cells inside the vascular niche, which are considered a natural reservoir of MSCs/pericyte lineage, are activated to migrate and differentiate toward myofibroblasts, under the influence of ECs [15,18]. In this context, we described that SSc-ECs strongly increased the expression of α-SMA and collagen-related genes, both in SSc- and HC-MSCs, which were already upregulated in SSc-MSCs. Of note, HC-ECs were able to downregulate both α-SMA and collagen genes in SSc-MSCs. Taken together, these results show the pivotal role played by pathological ECs from SSc patients in influencing the micro-environment, by factors released during the crosstalk with pericytes, and point out the pivotal role of dysfunctional SSc-ECs in influencing MSC differentiation (derived from patients or controls) toward myofibroblasts. These *in vitro* data suggest the hypothesis that endothelial cell dysfunction, which is a very early event, in SSc patients, may be a potential trigger leading to fibrosis, via the involvement of multipotent perivascular cells.

We showed that SSc-ECs constitutively expressed higher amounts of PDGF-BB and TGF-β. Furthermore, when SSc-ECs were co-cultured with SSc-MSCs or HC-MSCs they induced significantly increased production of both these molecules from MSCs. Recently, it has been shown that the overexpression of PDGF or TGF-β in normal mouse skin, engineered by adenoviral vectors, leads to pericyte detachment and vessel destabilization [41]. Furthermore, PDGF and TGF-β induced accumulation and expansion of connective tissue cells in the perivascular space, expressing both pericyte and fibroblast markers. Taken together, these data suggest that PDGF and TGF-β, produced by ECs play an important role in the generation and expansion of myofibroblasts, originating from microvascular pericytes [42].

Although PDGF and TGF-β promote vessel stabilization via pericyte progenitor recruitment and maturation during vasculogenesis in embryonic life, the same molecules during adult life might play an opposite role in reactive conditions after vascular injuries, thus, leading to vessel destabilization and myofibroblast generation [43].

The interaction between TGF-β and PDGF-R has been recently shown as a unique characteristic in scleroderma fibroblasts, which respond to TGF-β with upregulation of PDGF-R, making scleroderma fibroblasts more responsive to the subsequent mitogenic stimulation with PDGF [44]. In our experiments we observed constitutive upregulation of TGF-β in SSc-ECs. These cells were able to induce strong upregulation of PDGF-R on SSc-MSCs, when co-cultured together. These findings support the hypothesis that an intramural PDGF/PDGF-R loop, synergistic with TGF-β production, may cooperate in myofibroblast differentiation.

**Figure 6:**
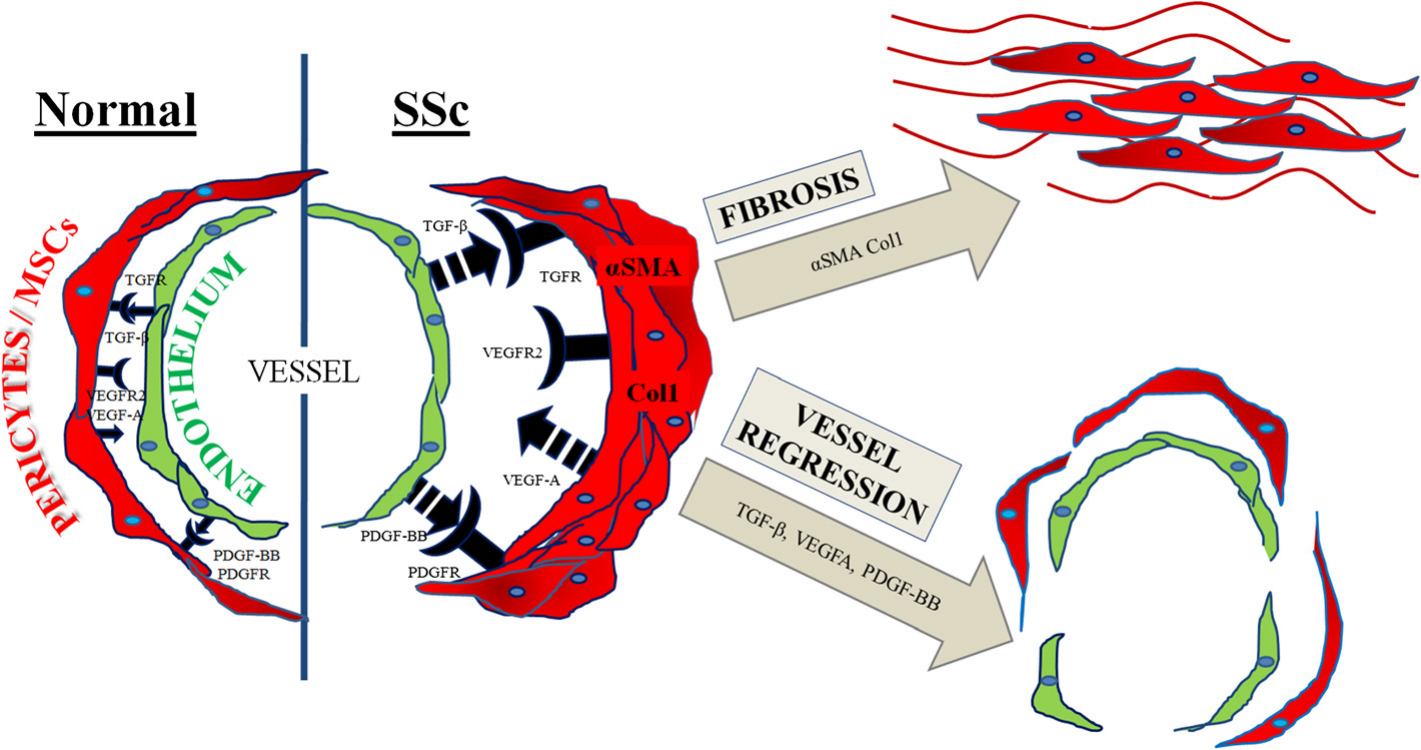
**Schematic drawing that illustrates the pathogenic consequences of altered crosstalk between epithelial cells (ECs) and pericytes/mesenchymal stem cells (MSCs) in systemic sclerosis (SSc).** In SSc, abnormal EC production of transforming growth factor (TGF)-β and platelet-derived growth factor (PDGF)-BB interacting with their specific receptors, expressed on pericytes/MSCs, may induce myofibroblast differentiation and activation, leading to the fibrotic manifestation of disease. Furthermore, the same molecular imbalance leads on one hand to pericyte proliferation and consequent wall remodeling in arteriolar vessels, on the other to pericyte detachment in capillaries. Furthermore, pericyte hyper-production of vascular endothelial growth factor (VEGF)-A may make ECs unresponsive to the proangiogenic stimuli of the tissue-derived form of VEGF-A. The increased expression of VEGFR2 on pericytes/MSCs may suppress PDGFR signaling through the induction of VEGFR2/PDGFR complexes. This pathway abrogates pericyte coverage and leads to vascular instability and regression. These alterations underlie the main pathogenic aspects of the SSc vasculopathy and the impaired compensatory neo-angiogenesis.

At present, emerging data suggest that MSCs are part of the perivascular niche, in which heterotypic cell-cell crosstalk between ECs and MSCs is able to regulate the perivascular cell fate [15]. This intimate interaction maintains MSCs in their quiescent state or provides signals toward differentiation, aiming to repair damaged tissues. Using a three-dimensional system for co-culturing ECs and MSCs, followed by cell sorting, we demonstrated altered crosstalk between MSCs and ECs in SSc, resulting in pathogenetically relevant changes in gene expression and protein levels of several molecules involved in the angiogenic and profibrotic processes which are schematically represented in Figure 6. These alterations lead to a phenotypic switch of perivascular multipotent cells toward migratory and profibrotic myofibroblasts, thus, linking the vascular damage to some fibrotic events underling the development and progression of SSc.

## Conclusions

Although our descriptive observational study does not directly address the mechanism of EC/MSC crosstalk, it gives us much information on the modulation and production of molecules involved in the pathogenesis of SSc, which may be targeted in future studies and suggests that a better understanding of the interplay between ECs and perivascular MSCs might open new future possibilities for regenerative medicine, targeting the early steps of fibrosis, for which convincing therapies are not presently available.

## Abbreviations

Ang1/2: angiopoietin1/2
BM: Bone marrow
Col1: collagen1
DMEM: Dulbecco’s modified Eagle's medium
ECs: endothelial cells
ELISA: enzyme-linked immunosorbent assay
FBS: fetal bovine serum
HC: healthy control
MSC: mesenchymal stem cell
PBS: phosphate-buffered saline
PDGF: platelet-derived growth factor
SSc: systemic sclerosis
TGF-β: transforming growth factor β
VEGF: vascular endothelial growth factor
αSMA: alpha-smooth muscle actin
